# Progress in the HIV epidemic: Identifying goals and measuring success

**DOI:** 10.1371/journal.pmed.1002729

**Published:** 2019-01-18

**Authors:** Jeb Jones, Patrick S. Sullivan, James W. Curran

**Affiliations:** Department of Epidemiology, Rollins School of Public Health, Emory University, Atlanta, Georgia, United States of America

## Abstract

Substantial progress has been made towards the goal of ending the HIV/AIDS epidemic due to advancements in both prevention and treatment of HIV. However, major challenges still remain. We describe basic principles of epidemic control in the context of HIV and identify a number of attainable goals in terms of control and elimination of HIV in specific populations and risk groups, given currently available HIV prevention and treatment methods. Currently available HIV prevention methods make it a feasible goal to eliminate HIV transmission attributable to mother-to-child transmission and blood transfusions. Reductions in transmission attributable to sexual behavior and injection drug use are feasible, but elimination of these modes of transmission will require further advancements in behavioral and biomedical HIV prevention. With regard to HIV-related mortality, we argue that elimination of death due to HIV-related causes is a feasible goal. HIV-related deaths should be treated as sentinel events triggering epidemiological investigation into the breakdowns in the HIV care continuum that led to them. We briefly discuss additional considerations that will affect the success of HIV prevention programs.

## Abstract 2

In a Collection Review, Jeb Jones and colleagues discuss goals, practicalities, and metrics in efforts to end the HIV/AIDS epidemic.

## Introduction

The United Nations has declared a goal of ending the AIDS epidemic by 2030 [1], an aspiration echoed by many individual countries. To achieve this goal, targets have been set for each step in the HIV diagnosis and care continuum. Specifically, besides primary prevention targets for voluntary medical male circumcision (VMMC) [2,3], pre-exposure prophylaxis (PrEP) [4,5], use of condoms [6], harm reduction [7], and opioid substitution treatment [8], the 90-90-90 goals aim for 90% of individuals infected with HIV to be aware of their status, 90% of those diagnosed to initiate antiretroviral (ARV) treatment, and 90% of those on ARVs to have viral loads suppressed below levels of detection, by 2020 [9,10]. These aspirational goals are laudable and, to the degree that they can be achieved, will have a meaningful impact on HIV incidence as well as mortality globally. However, the heterogeneity of the HIV epidemic globally raises challenges in terms of measuring success along the way. In a recently published article, Peter Ghys and colleagues have proposed six different metrics to measure transitions in different aspects (e.g., incidence, mortality) of the HIV epidemic [11].

Differing definitions of what it means for the epidemic to be “over” or “under control” can lead to confusion among stakeholders and the public. It is crucial to define quantifiable goals that will be clearly understood and can be used to measure progress toward vanquishing HIV/AIDS as a major public health problem. This will bring focus and provide benchmarks to measure success of HIV prevention and care programs globally. The purpose of this paper is to review concepts of epidemic control and apply them to the case of HIV. We identify aspirational but feasible goals for control and elimination of HIV transmission and HIV-related mortality. Finally, we will briefly discuss additional challenges and considerations that play an important role in controlling the HIV epidemic.

## Epidemiological principles

Disease occurrence is typically measured in terms of incidence (all new cases of a disease during a given period of time) and prevalence (all existing cases of disease at a given point in time). Incidence and prevalence represent measures that we aspire to accurately count or estimate to characterize a certain disease at the population level. However, there are practical considerations, particularly with respect to HIV, that make estimating these measures difficult.

HIV incidence is difficult to measure because HIV infections are initially asymptomatic or cause minimal nonspecific symptoms. Therefore, most newly infected persons do not immediately seek HIV testing and are often diagnosed many months or years after infection. In addition, HIV infection is associated with a window period of one to three months, during which antibody tests cannot detect infection, meaning that early infection may be missed, even when people with very recent HIV infections are tested for antibodies to HIV. Because of these challenges, new infections are frequently measured in terms of new HIV diagnoses. Although this is a pragmatic approach, reporting diagnoses instead of incidence can be problematic in terms of understanding prevailing disease dynamics. For example, increases in diagnoses might be due to more effective HIV testing campaigns that identify previously undiagnosed individuals. The proportion of new diagnoses that occur among individuals with CD4 cell counts <350 (i.e., late-stage diagnosis) highlight the fact that many new diagnoses are not new cases of HIV. For example, a 2015 Dutch study found that 28% of men who have sex with men (MSM) had a CD4 count <350 at the time of diagnosis [12]. Similarly, the proportion of late-stage diagnoses in a 2013 South African study was 34% [13]. It is a goal to reduce the time between HIV infection and HIV diagnosis in order for persons to access ARV treatment and to reduce incident infections. As an alternative to using new HIV diagnoses as a proxy for HIV incidence, statistical modeling methods can be used to obtain estimates of HIV incidence in general and within specific populations [14,15].

In the absence of curative therapy, HIV is a lifelong infection. Thus, prevalence measures alone cannot provide a meaningful indication of changes in the epidemic in the short or near term; changes in prevalence must be interpreted in the context of HIV incidence, treatment outcomes, and mortality. Reducing HIV transmission and incidence requires identifying and counselling persons with HIV infection and providing lifelong ARV treatment in order to decrease the probability of transmission of the virus and reduce HIV-associated mortality, in addition to increasing coverage of primary HIV prevention methods among those at risk of infection. Programmatically, these are costly endeavors; estimates suggest that the cost of mounting a comprehensive AIDS response will be US$26–$36 billion annually [1,10,16].

Standard measures of epidemic control include control, elimination, eradication, and extinction (**Box 1**) [17,18]. These measures represent increasing levels of success with respect to ending disease incidence. The language surrounding epidemic control can be confusing, because the terms describing each level of control have common and imprecise meanings in colloquial language. In this paper, we use the words control, elimination, eradication, and extinction only in accord with their technical definitions.

**Box 1. Relationship between incidence, prevalence, and duration of disease under steady state**

Prevalence = Incidence * Average duration of disease

This equation demonstrates that reductions in prevalence can be achieved by reducing incidence of a disease or the average duration of a disease. Conversely, increases in incidence or duration will increase prevalence. This is why public health successes, such as increasing coverage of ARV therapy, which increases the average duration of HIV infection by extending the life span, leads to increases in HIV prevalence.

**Measures of disease control**

Control: reduction of disease incidence, prevalence, or mortality in a geographically defined area to a locally acceptable level via effective interventions.

Elimination: complete cessation of incidence in a geographically defined area. Because the disease-causing agent persists, elimination requires ongoing intervention to be maintained.

Eradication: complete removal of the disease-causing agent from the natural environment. The disease-causing agent might persist in controlled laboratory environments. Prevention interventions are no longer needed.

Extinction: complete removal of the disease-causing agent from all natural and laboratory environments.

## Formulating goals for controlling the HIV epidemic

HIV has unique characteristics that affect the interpretation and utility of standard measures of epidemic control. Any measurable goals that are put forward in terms of reducing incidence of HIV must take into account the biological and sociological characteristics of the epidemic. Because eradication requires that a disease-causing agent is entirely removed from the natural environment on a global scale, eradication of HIV will not be an attainable goal until a vaccine and cure are developed and uniformly utilized. **Table 1** presents aspirational epidemic control goals in terms of HIV transmission and HIV mortality that are attainable using current prevention methods. Control of the HIV epidemic includes both primary prevention of HIV among those at risk of infection and treatment of persons living with HIV (PLWH) to prevent HIV-related mortality and reduce the risk of onward transmission. Although these are distinct processes, there are some overlapping approaches that contribute to both.

**Table 1:** Aspirational but feasible goals for control and elimination of HIV incidence and HIV-related mortality based on existing methods.

Control		
	HIV Transmission	HIV Mortality
Modes of Transmission	Sexual Injection drug use	N/A—recommend goal of elimination (rather than control) for HIV mortality
Tools	PrEP Condom use VMMC HIV testing Couples counseling and testing ARV treatment and viral suppression Needle exchange programs Opioid substitution programs	
Barriers	Stigma Behavior change Silent infections Insufficient resources	
Elimination		
	HIV Transmission	HIV Mortality
Modes of Transmission	Mother-to-child Blood transfusion associated	N/A
Tools	Prenatal HIV testing ARV therapy Safe baby formula Blood supply HIV testing	HIV testing ARV therapy
Barriers	Awareness Resources Lack of availability of safe alternatives to breastfeeding in some populations	Stigma Resources Adherence Complementary services needed to support adherence and treat comorbid conditions such as substance abuse, tuberculosis, and hepatitis
Comments	Uniquely feasible opportunities to completely eliminate HIV incidence attributable to these two modes of transmission	N/A

Abbreviations: ARV, antiretroviral; N/A, nonapplicable; PrEP, pre-exposure prophylaxis; VMMC, voluntary medical male circumcision.

### HIV transmission

Elimination achieved within a given geographic region by mode of transmission is defined as a reduction of incidence to zero. Unlike with eradication, the disease-causing agent continues to persist in the population, and prevention efforts are necessary to ensure that elimination persists. Elimination of incident infections could thus theoretically be achieved even as HIV prevalence persists, using existing prevention methods like treatment as prevention [19], PrEP [4,5], condoms [6], VMMC [2,3], harm reduction [7], and opioid substitution programs [8], but only if these interventions are universally and continually utilized over time. Thus, goals related to transmission differ by risk populations.

The aspirational goal of elimination of transmission is more feasible for transmission attributable to blood transfusions and mother-to-child transmission because prevention methods are currently available that are very effective [20,21], and because these modes of transmission largely occur within medical settings, in which interventions can be scaled up universally. HIV infections caused by blood transfusions can be eliminated by testing all donated blood. Mother-to-child transmission can be eliminated by HIV testing all pregnant women, by providing lifelong ARV therapy to mothers who are diagnosed, by providing appropriate prophylaxis at the time of birth, and by following the breastfeeding guidelines established by the World Health Organization [22]. Transmission events due to these causes should be investigated to understand where the breakdown in systems to identify mothers living with HIV and systems to protect infants occurred, so that necessary systems and policies can be put in place to ensure universal coverage of these prevention methods. Of note, new HIV infections among children had reduced 35% to 180,000 in 2017 compared to 270,000 in 2010 [23].

HIV transmission due to sexual behavior and injection drug use can be controlled, but elimination is not currently realistic in that the number of transmissions is considerably higher and the structural opportunities for intervention (such as in the context of perinatal care) do not exist. A realistic goal will therefore be epidemic control (e.g., to reduce HIV incidence to locally acceptable low levels, which should be defined clearly and represent considerable improvement over the status quo). Many prevention tools, both old and new, are available to reduce the transmission of HIV due to these risk behaviors. The effectiveness of ARV therapy [19] and PrEP [4,24]—in addition to condom use [25,26], needle and syringe exchange programs [27], and VMMC [2,3]—in reducing the risk of HIV transmission following strong adherence by HIV^+^ and HIV^−^ individuals, respectively, demonstrates that existing methods have the potential to greatly reduce HIV transmission. Delivering these tools in combinations and in appropriate service settings, monitoring the uptake in target populations, and monitoring for inequities in uptake will be important to maximize impact. Current ARV-based prevention strategies require substantial levels of adherence for prevention efficacy; these tools can be improved by considering long-acting formulations or shorter, on-demand approaches. A safe and effective vaccine and curative therapy, neither of which are currently available, would change the HIV prevention and treatment landscape. These types of future advancements in biomedical and behavioral prevention research are needed to achieve a goal of elimination of sexual and injection drug use transmission.

### HIV mortality

Given current treatment options, elimination of HIV mortality should be an aspirational yet feasible goal. Combination ARV therapy with substantial adherence has been shown to be effective in reducing viral loads and extending the life span of PLWH [28]. Expanding testing programs to identify all PLWH followed by treatment initiation and adherence support for those persons who are diagnosed could lead to the elimination of mortality due to AIDS-related complications. Deaths from HIV-related causes should be treated as sentinel events, instigating a public health investigation to understand the factors leading to treatment failure. In 2017 there were approximately 940,000 AIDS-related deaths globally [23]. Appropriate resources will need to be made available to investigate the complex, multilevel failures that likely contribute to HIV-related mortality, including limited healthcare access, poverty, substance use, and mental health problems.

## Current progress in controlling the HIV epidemic

Since the 90-90-90 targets were announced in 2014, substantial progress has been made. Compiling country-level data from around the world, the Joint United Nations Programme on HIV/AIDS (UNAIDS) has summarized progress through the end of 2017 [29]. Although progress—in some countries, substantial progress—has been made, a number of gaps and challenges remain. It is beyond the scope of this paper to fully review global progress, but there are some conspicuous trends. Gains in epidemic control have been notably slower among men and young people, highlighting that existing interventions and programs are not equally effective across demographic groups. This is evident in the faster declines in mortality from AIDS-related causes among women compared with men since 2000. This suggests that additional work will be necessary to increase uptake of HIV testing and treatment among groups that lag behind in the current environment. Additional marginal gains towards the 90-90-90 targets will require engaging these harder-to-reach groups.

There have also been consistent increases in HIV infections and AIDS-related deaths in Eastern Europe and Central Asia since 2000. This is in contrast to generally stable or declining rates of HIV infections and AIDS-related deaths in other regions globally. This suggests additional dynamics that need to be understood and managed in Eastern Europe and Central Asia, in order to halt the growing epidemic in this region.

## Varying definitions of control: Defining feasibility

Control of the HIV epidemic should be measured at the local, national, and global levels. To achieve control of the HIV epidemic will mean identifying local goals that result in forward progress but are achievable for a given epidemiological context, as well as identifying and implementing the means to achieve those goals. In the 2016 Prevention Gap Report, UNAIDS identified a target of fewer than 500,000 new HIV infections globally by 2020 [30]. To achieve this goal, the report recommends implementing the Fast-Track strategy, including achieving the 90-90-90 targets in combination with primary prevention programs. Substantial progress will be required to achieve the 90-90-90 targets globally by 2020, and, under this aspirational goal, a substantial proportion, that is 27% of PLWH, will continue to have unsuppressed viral loads (i.e., if the goals are met, about 73% of PLWH will have a suppressed viral load [90% * 90% * 90% = 73%]).

Beyond identifying methods most appropriate to measure successes and identify challenges in HIV prevention, defining epidemic control also requires identifying aspirational yet feasible goals for a given geographical region and epidemiological context. Because control is achieved within a specific geographic region, the local dynamics and epidemic characteristics must be considered in order to identify appropriate goals. Thus, although benchmarks such as the 90-90-90 goals are helpful in planning at a global level, the target epidemiologic measures of incidence, prevalence, transmission rate, or mortality might need to be different depending on local epidemic characteristics. Goals must be identified in terms of what is aspirational, feasible, and measurable at the local, national, and global levels.

## Additional considerations

Any discussion of epidemic control must acknowledge the sociocultural context in which HIV exists. It will be difficult, if not impossible, to achieve control of the HIV epidemic without addressing other factors that contribute to the epidemic, such as stigma and criminalization associated with HIV infection and the need for ancillary health services.

Stigma associated with HIV infection and HIV risk behaviors continues to pose a challenge to public health programs designed to reduce HIV incidence [31]. Stigma can reduce the likelihood that an individual is aware of their status and seeking treatment or effective prevention tools owing to fear of testing and disclosure. Joseph Amon and colleagues [32] have described the importance of measuring stigma in addition to other disease-specific indicators (e.g., HIV incidence, AIDS-related mortality) in order to track changes in the epidemic over time.

HIV prevention and treatment programs exist within larger healthcare and economic systems and PLWH, and those at risk of HIV seroconversion, have health needs beyond HIV treatment and prevention services. Mental health services, drug treatment programs, and programs ensuring continuity of care for incarcerated populations are all necessary components of a holistic approach to HIV prevention. Furthermore, these issues serve as a reminder that the populations of PLWH without a diagnosis and those at high risk of HIV infection are very heterogeneous, and there are additional challenges that can be barriers to reaching them. As programs approach the 90-90-90 goals, continued progress might be increasingly challenging because certain key populations have not been reached in individual settings, or different strategies might be required to make further progress.

## Conclusions

In this paper, we have reviewed concepts related to epidemiological control in the context of the biology and epidemiology of HIV infection and considered sociobehavioral factors that contribute to the HIV epidemic. Substantial progress has been made in the past 35 years with regard to HIV prevention, treatment, and surveillance methods; however, significant challenges remain. The global commitment to ending the HIV epidemic, exemplified through the United Nations’ *Political Declaration On HIV and AIDS* [1], indicates a high degree of motivation from countries around the world to reduce morbidity and mortality due to HIV/AIDS. Existing strategies to measure and evaluate progress of HIV prevention interventions should be examined, and additional new measures should be considered in order to most effectively characterize current and future progress in stemming the epidemic. Targets related to controlling HIV incidence and AIDS-related mortality should reflect realistic capabilities of currently available interventions.

In addition to reliable data to track progress in controlling the HIV epidemic, continued research, including implementation science, will be needed to further our understanding of the most effective HIV prevention interventions and how to most effectively deploy these interventions. As outlined above, we identified elimination of HIV transmission due to mother-to-child transmission and blood transfusions as currently attainable. In order to meet this goal, however, resources will need to be allocated appropriately to ensure that appropriate interventions are broadly available, and studies will need to be conducted to understand barriers to implementation when those interventions are underutilized or ineffective. A holistic approach including surveillance, appropriate measures to track epidemic transition, and implementation science is key to continued progress in meeting global targets for control of the HIV epidemic.

## Abbreviations

ARV: antiretroviral
MSM: men who have sex with men
PLWH: persons living with HIV
PrEP: pre-exposure prophylaxis
UNAIDS: Joint United Nations Programme on HIV/AIDS
VMMC: voluntary medical male circumcision
